# Chikungunya and diabetes, what do we know?

**DOI:** 10.1186/s13098-018-0329-2

**Published:** 2018-04-13

**Authors:** Francisca Kalline de Almeida Barreto, Renan Magalhães Montenegro, Virginia Oliveira Fernandes, Rhaquel Oliveira, Lívia Aline de Araújo Batista, Akhtar Hussain, Luciano Pamplona de Góes Cavalcanti

**Affiliations:** 1 Federal University of Ceará (UFC), Rua Professor Costa Mendes, 1608, Rodolfo Teófilo, Fortaleza, CE 60416-200 Brazil; 2 Department of International Health, Institute of Health and Society, Faculty of Medicine, University of Oslo, P.O. Box 1130, Blindern, N-0317 Oslo, Norway

**Keywords:** Chikungunya, Diabetes mellitus, Arbovirus, Diabetes complications

## Abstract

**Background:**

Chikungunya (CHIK) is a viral disease transmitted by mosquitoes. The first cases in Brazil were confirmed in 2014. Between 2016 and 2017, over 300,000 cases were identified during this period, with nearly 300 deaths. The clinical manifestations, pathogenesis and risk factors for occurrence of severe cases are not yet well understood, although it is known that the severity of the cases is associated with the presence of comorbidities, especially diabetes mellitus (DM).

**Objective:**

To review the medical literature for the associations between DM and CHIK and to understand the potential impact on metabolic state and its complications.

**Methods:**

Literature review was carried out to search for articles (English, Portuguese and Spanish) in Medline and Virtual Health Library databases for the period between 1952 and 2017, with the following keywords: “Chikungunya fever”, “Chikungunya virus”, “diabetes mellitus”, “diabetes”, “diabetes complications “and “multi-morbidities (MeSH) “with interposition of the Boolean operator “AND”.

**Results:**

After removal of duplicities and following exclusion criteria, 11 articles were selected. Our results showed that the patients of CHIK with DM had more severe and prolonged symptoms of CHIK and more frequently required hospitalization. No study investigated the biological process to explain how hyperglycemic state worsened the clinical manifestations of Chikungunya in diabetic patients.

**Conclusion:**

An important association between DM and the severity of CHIK is observed. Prospective and more rigorous controlled studies are required to generate evidence that might y elucidate the causes of this relationship. Given the fast expanding viral infection of Chikungunya in Central and South America, Asia and Africa in recent years in the context of exponential increase in diabetes globally, the issue deserves global attention.

## Background

The Chikungunya virus (CHIKV) is an *Alphavirus*, belonging to the *Togaravidae* family [1]. It was first described in 1952, in the South of Tanzania [2]. About 2.9 million of suspected cases of Chikungunya (CHIK) were recorded in Central America, North and South America, with 296 deaths attributed to the condition in 2016 [3, 4]. In Brazil, between 2016 and 2017 more than 300,000 cases have been confirmed with nearly 300 deaths [5].

After the introduction of CHIKV in a region infested by mosquitoes it is estimated that up to 70% of the population may be infected [6]. The condition has a clinical course that may vary from asymptomatic infection to serious and potentially fatal illness [7, 8]. The risk factors for chronification or illness severity are not clear, but they seem to be associated with the presence of comorbidities. In this scenario, more severe illness was found to be related to the preexistence of diseases like diabetes mellitus (DM) [9]. The DM is a serious health issue with more than 425 million cases diagnosed in the world [10].

Since most CHIK deaths occurs in population aged 60 years-old and above, and the prevalence of metabolic diseases, such as DM is also highly prevalent in this age group, scientist have been trying to find a correlation between these conditions Serious infectious diseases, like CHIK, are known to disturb metabolic regulation. DM may alter the immune response but also health status may be more compromised by DM and/or aging in patients with CHIK.

A systematic literature review was performed to explore existing data for the peculiarities of CHIKV infections in individuals with DM.

## Methods

### Inclusion and exclusion criteria

This systematic review has adapted the recommendations of Preferred Reporting Items for Systematic Reviews and Meta-Analyzes (PRISMA) guidelines. Full text studies were selected, regardless of the methodological approach, published in English, Portuguese or Spanish. Duplicate studies were excluded and those containing only one of the themes (for example only Chikungunya) and studies who were not performed in humans.

### Search strategy and review procedures

We searched for articles published from January 1952 to August 2017 in the following databases: National Library of Medicine (NLM), which uses PubMed, and in the Virtual Health Library (VHL/BIREME), which includes LILACS, IBECS, Medline, Cochrane Library and SciELO. The search in the electronic database was held in August and September 2017, through advanced search using as controlled descriptors (Medical Subject Headings, MeSH): “Chikungunya fever”, “Chikungunya virus”, “diabetes mellitus”, “diabetes”, “diabetes complications”, “comorbidities”, and “ multimorbidity (MeSH) with interposition of the Boolean operator “AND”.

The articles identified by the search strategies were independently evaluated by two researchers. The first analyzes were based on the reading of the titles, abstracts and keywords, and after the complete article using the inclusion criteria. In cases of disagreement, a third evaluator was consulted to reach consensus.

The guiding question of review was: what is the scientific evidence available in the literature about the association between diabetes mellitus and Chikungunya? Results of the systematic analysis were presented in three chapters.

## Results

**Fig. 1:**
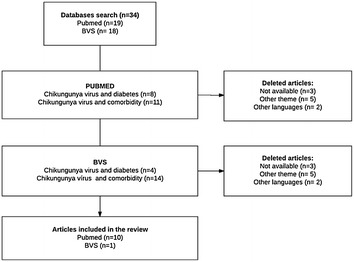
Studies of selection flowchart

Thirty-four (34) articles were selected, of which four (4) were excluded because of the language, eight (8) for not dealing with diabetes mellitus, Chikungunya infection or comorbidities and two (2) were not available. Other 12 were excluded for duplication (Fig. 1). In the initial step, 11 full articles were found (Table 1).

**Table 1:** Selected articles for the study and main findings

Year	Author	Journal	Study design	Main finding
2007	Borgherini et al.	Clin Infect Dis	Cross-sectional	Study of 157 patients with acute CHIK, showed that 52 of them had diabetes mellitus (DM) as comorbidity. Most of them were over 45 years of age, of these, 41% required hospitalization due to worsening of the viral infection, giving a significant higher rate of hospitalization OR (p = 0.008, OR 2.8 CI 1.32–5.94)
2009	Economopou-lou et al.	Epidemiol Infect	Cross-sectional	The study included 610 cases with an incidence rate of atypical CHIK 112/100,000 inhabitants, of whom 89% had associated comorbidities. DM was the second most reported comorbidity (39%). Further the results also showed that, of the total cases, 131 patients presented glycemic imbalance and 27 (20%) of them were diagnosed with DM for the first time. Of the 147 severe atypical cases with neurological disorders, 24% were diabetic. And those patients with severe cardiac conditions (n = 226), 48% had diabetes. Patients aged 40–60 years had a 2.5-fold higher risk of developing atypical forms of CHIK, while older patients 60 years and above had 1.6-fold higher risks in addition
2009	Staikowsky et al.	PLoSOne	Prospective	Patients with acute CHIKV were divided into two groups: active viremia and no viremia. It was observed that patients with active viremia had more comorbidities, mainly DM = 44/180 (24.4%) as compared to 8/34 (33.3%) without viremia. Female patients had frequent comorbidities (p < 0.05) as opposed to male patients. It was also observed that patients who required hospitalization were older and had more comorbidities (p < 0.001)
2009	Tandale et al.	J ClinVirol	Prospective	The study described cases of CHIK patients in two cities classified as classic, severe, and severe with neurological damage. Of 149 patients, DM was present only in severe cases (4/25) and in severe cases with neurological damage (4/10 and 15/59)
2009	Sissoko et al.	PLoSNeglTropDis	Restrospective cohort	The search for rheumatic manifestations in patients with CHIK were followed retrospectively. The results showed that of 147 patients confirmed with CHIKV, 32 had DM as comorbidity (OR 2.3, 0.9–5.3). In addition, the study showed that 84 patients remained with symptoms of rheumatic arthritis. Comorbidity was more common in these patients (OR 3.0, 1.5–5.9)
2012	Couturier et al.	Rheumatology	Prospective cohort	This was a prospective cohort analysis of the quality of life of patients with CHIK. Results showed that of 377 patients, 227 (60%) had associated comorbidities, OR 0.71 (0.57, 0.88), p > 0.002. Of these, 23 had DM
2015	Tolokh et al.	Am. J. Trop. Med. Hyg	Case report	Case report of a 50-year-old Puerto Rican man with diabetes with acquired acute Chikungunya virus infection. The patient evolved with atypical characteristics and severe manifestations such as leucopenia, thrombocytopenia, increased glycemic levels and ketone bodies, hepatic enzyme alterations, which culminated in the developmentof the diabetic ketoacidosis
2016	Crosby et al.	Int J Infect Dis	Cross-sectional	In a cross-sectional study of 65 patients with severe CHIKV admitted to Intensive Care Units, 54 had comorbidities (83%); DM wasthe second most prevalent comorbidity (32%). The study also showed that most diabetic patients did not have classic symptoms of Chikungunya, but may hadmore severe forms of the disease (p = 0.01)
2016	Jean-baptiste et al.	Am. J. Trop. Med. Hyg	Cohort	The only study that showed a direct relationship of DM in hospitalized patients with CHIKV, in the acute phase of the disease. It was found that diabetic patients worsened CHIK condition (p < 0.002) and presented greater arthralgia, fever and myalgia than non-diabetic patients (p < 0.008). Only diabetes was identified as a significant contributor to the presence of the triad of arthralgia, fever and myalgia (p = 0.002), time to improve arthralgia (p < 0.001) and duration of fever (p = 0.002). Diabetic patients had longer hospital stay (p < 0.0001) and a mean increase in glycaemia of 26.8% (p < 0.0001) when compared to baseline. Forty percent of diabetic patients required adjustment of the drug dose during the course of infection
2016	Perti et al.	Plos	Cross-sectional	The study reported that of 180 patients with CHIK, 71 had DM, and these patients had a higher risk for hospitalization, due to worsening of the condition (RR: 1.53; CI 95% 1.15–2.03). When adjusted for age the risk was 1.39 (1.06–1.84) (p = 0.02)
2016	Rollé et al.	EmergInfectDis	Cross-sectional	Diabetes mellitus was the most cited comorbidity in patients with severe CHIK 16/42 and non-severe CHIK 28/68

### Association between Chikungunya and Diabetes

Our findings suggest that the clinical condition deteriorated for patients with Chikungunya when comorbidities were present. diabetes mellitus was found to be the second most cited comorbidity after arterial hypertension [11–17].

Diabetes has always been found as one of the main comorbidities associated with greater severity of symptoms of CHIK. Besides, but it is also associated with a higher rate of hospitalizations (RR: 1.39; 95% CI 1.06–1.84). Common causes of hospitalizations were: presence of unstable vital signs, as tachycardia; an increase in the number of abnormal laboratory results, as leukocytosis; acute kidney injury and liver enzyme abnormalities [11, 14, 18].

### Clinical presentation of Chikungunya in diabetic patients

One study with 169 patients infected with CHIKV showed that the presence of diabetes has worsened the severity of symptoms and changes in glycemic state resulting in necessary adjustments for medications in approximately 40% of the cases, even in those with insulin treated patients. Further, DM was found as a significant contributor for the presence of arthralgia, fever and myalgia (p = 0.002) and a longer time required for the improvement of arthralgia (p < 0.001) and duration of fever (p = 0.002) [19].

Another study with atypical cases of CHIK showed that a higher number of patients were diagnosed with diabetes for the first time. Besides, DM was present in higher rate in patients with higher fever peaks, cardiovascular diseases and severe neurological disorders [9].

### Metabolic control in diabetic patient with Chikungunya infection

A case report showed that a diabetic patient who acquired acute CHIK infection presented worsening of glycemic control and developed ketoacidosis. The case presentation included fever, weakness, diffuse myalgia, nausea and several episodes of diarrhea, as well as lymphopenia, thrombocytopenia and elevated liver function, required hospitalization and intensive care [20].

Another study found that most patients with acute CHIK who required intensive care had pre-existing comorbidities, including diabetes mellitus [11]. Studies have also shown changes in glucose metabolic rates in patients with CHIK [19].

## Discussion

Diabetes mellitus is an important comorbidity, associated with severe cases of Chikungunya. The infection alters glucose levels in diabetic patients, although there are scarce literature data. The most important findings in patients with hyperglycemia were significant worsening of symptoms, which implies greater morbidity in these patients when compared to patients without diabetes. In addition, the time of improvement for symptoms were longer in these and required more intensive care. It is important to note that the diabetic patients with CHIK presented a poor glycemic control, requiring adjustments in DM therapy. Further, these patients developed acute complications of diabetes. However, the pathophysiology of this association remains unknown. It is not known whether hyperglycemia changes the virulence of infection, or if the virus modifies the glycemic metabolism. In addition, the impact of treatment for CHIK on glucose regulation remains unspecified.

Diabetic patients are more susceptible to infection, and this can impact on glucose metabolism [21]. Probably, this stress aggravates insulin resistance in these cases which justify the metabolic impact of CHIKV [22]. In addition, DM is not just a disorder of glucose metabolism, but a chronic inflammatory condition characterized by multiple changes in lipid profiles and blood glucose [23]. Such inflammatory processes are due to hyperglycemia which leads to increased synthesis of glycosylation end products (AGEs), activates macrophages and other cells of the immune system, increase oxidative stress and promote the synthesis of pro-inflammatory cytokines, besides stimulating the synthesis of adhesion molecules that facilitate inflammation in the tissues [24]. This worsens the patient conditions and increases rates of complications, including vascular diseases, renal diseases and neuropathies [25]. The inflammatory process and its complications might provide a higher propensity to infections or a greater severity of these conditions. This was evidenced in studies involving dengue virus in diabetic patients, which showed that patients with DM had a higher risk of developing severe dengue [26, 27]. Another study with diabetic patients and the West Nile virus had observed that these patients were at greater risk of developing encephalitis and other severe forms of the disease [28]. Both are arboviruses with features similar to CHIKV, and may be related to the mechanism of pathogenesis, although it was not clear how the diabetes worsens the clinical presentation of these infections.

A hypothesis that could explain this mechanism would be that the CHIKV disrupts the metabolism of important cytokines, weakening the immune system. As shown in a study, there is a defect or malfunction of the type I interferon (IFN-I) in patients with diabetes, which would cause a decrease in response to CHIKV and, as a result, would increase the viral loads and the severity of the disease [29]. Several studies have identified the importance of innate host immune responses, particularly the response of IFN-type I, for the control of alphavirus infections with tropism for joints [30].

Another important issue is how this inflammatory and immune response occurs in diabetic patients, as well as whether the virus itself interferes with insulin secretion or the glycemic control. Following this line of reasoning, one study, by analyzing the metabolism of various proteins after inoculation of the CHIKV, found that some of the altered proteins were involved in lipid metabolism and glucose [22]. Such data suggest that the virus modifies the action of these proteins in the body, resulting in a negative impact of metabolic control. Despite these evidences, it is still unclear the developed of the pathophysiological process of this association.

New hypotheses are required to explain the causal relationship between DM and clinical presentation of CHIKV infection, as well as biochemical tests to clarify the molecular physiopathology involved with this two conditions.

There are no data regarding the correct management of diabetic patients who are affected by CHIK, and patients with CHIK who present glycemic decompensation. The reports about the management of patients with these conditions are sporadic. Therefore, due to lack of data and evidence-based knowledge, this clinical approach remains a challenge.

The studies published so far indicate that there is greater severity of cases of CHIK in patients with diabetes. Furthermore, an important increased in blood glucose levels was observed in patients with newly diagnosed diabetes who have CHIKV infection.

Furthermore, the number of deaths attributable to DM also increased by 35.2% in April 2017 during the outbreak of CHIKV in the Northeast of Brazil when compared to historical data (2001–2016). During the same period, 4394.4 cases of CHKIV registered per 100,000 inhabitants. This sudden increase in the number of deaths attributed to a chronic non-transmissible disease led to conjecture of the manifestation of an acute factor with the CHKV outbreak as an important element [31].

However, the most common limitation in selected studies was the use of sample per convenience and old data. Another important aspect that deserves attention is the complexities in the association of some deaths in patients with CHIK given the time of illness until death and the inherent difficulties in the investigation of these cases that affect primarily the elderly population [8]. It may also be that some studies have been published in non-accessible languages, since most of the cases prior to the Reunion Islands epidemic were in Africa, as part of the gray literature, and therefore, were not reached by the search for this review.

## Conclusion

Diabetes mellitus is an important comorbidity to the worsening of the clinical manifestations of patients with Chikungunya infection. This associations of CHIK and DM are of substantial public health importance and deserve proper attention, because both of them occurs largely in the elderly population. Even though the exact biological mechanism of the association is not known yet due to the limited available data, the association between CHIK and DM related to the severity of cases, complications and glucose deregulation are well documented.

Due importance of the diseases complexity and considering both, DM and CHIK, as a health threat in the world nowadays, is urgently needed a prospective well-designed study to elucidate the biological mechanism and the best clinical management of this association.

## Abbreviations

CHIK: Chikungunya
CHIKV: Chikungunya virus
DM: diabetes mellitus
NLM: National Library of Medicine
VHL: Virtual Health Library
BIREME: Latin American and Caribbean Center on Health Sciences Information
IBECS: Bibliographical Index of Health Sciences
AGEs: glycosylation end products
IFN-type I: type 1 interferon
